# Is There a Long-Term Link Between Digital Media Use and Adolescent Headaches? A Longitudinal School-Based Study

**DOI:** 10.3390/children11121549

**Published:** 2024-12-20

**Authors:** Clarissa Humberg, Verena Neß, Lisa-Marie Rau, Julia Wager

**Affiliations:** 1 German Paediatric Pain Centre, Children’s and Adolescents’ Hospital Datteln, 45711 Datteln, Germany; c.humberg@deutsches-kinderschmerzzentrum.de (C.H.); v.ness@deutsches-kinderschmerzzentrum.de (V.N.); l.rau@deutsches-kinderschmerzzentrum.de (L.-M.R.); 2 Department of Children’s Pain Therapy and Paediatric Palliative Care, Witten/Herdecke University, Faculty of Health, School of Medicine, 58448 Witten, Germany

**Keywords:** social media, video games, headache, pediatrics, multilevel analysis, screen time, school

## Abstract

Background/Objectives: The use of digital media, and especially social media, has been increasing over recent years. Previous research has reported a negative impact of media use on headaches; however, most of these studies are cross-sectional. Therefore, we conducted a longitudinal study to explore the relationship between different types of media usage (watching videos, gaming, and social media) and headache frequency and headache intensity over time. Methods: School-aged children from five German schools completed five assessments between 2017 and 2018. In total, *N* = 575 (72.9% female; *M*_age_ = 13.3, *SD*_age_ = 1.86) children and adolescents reporting consistent headaches across all assessments were analyzed. Multilevel linear modeling was used to assess the relationships between media use and headache frequency and intensity over time. Results: There were only minor associations between media use and headache intensity or frequency. Notably, only high social media usage was linked with worse headache intensity (*t*(1989) = 4.109, *p* < 0.001). Conclusions: The impact of media use on headaches seems to be less harmful than previous research might suggest. We believe that increased time spent consuming media should not be considered a risk factor for pain conditions but rather a helpful resource for pain management.

## 1. Introduction

Digital media has become an important part of daily life for children and adolescents. Over the years, a strong increase in digital media consumption has been observed in this age group [1,2]. Social media, in particular, is widely used and can offer numerous benefits, such as fostering connectedness, reducing feelings of loneliness, and enhancing social capital [3]. However, adverse physical and psychological health effects resulting from digital media use have also been reported [4,5,6]. Primarily, excessive media use has been correlated with negative outcomes like stress, depression [7], and pain conditions [8].

During the coronavirus disease 2019 (COVID-19) pandemic, media use among youth received significant public attention, highlighting the need to understand its impacts. Children and adolescents spend a considerable amount of time on screens, both for school-related and leisure activities. At the same time, the negative associations between digital media and adolescent mental and physical health have become more evident. Some studies have found that digital media adversely affects adolescent mental well-being, with most studies focusing on social media use [9,10,11]. Social media appears to be a risk factor for depression, addiction, and anxiety, among other conditions [9,10,11]. However, study results are mixed, often of correlational nature, and generally show weak effects [12,13].

The increase in digital media use also coincides with an increase in headache prevalence in adolescents [8,14,15]. In Germany, headaches are the leading cause of pain among adolescents, with around 37% suffering from headaches at least once a month [16,17].

Several studies have investigated the potential correlation between digital media consumption and headaches, finding positive associations, such as screen-based behavior (e.g., watching television) increasing the odds of experiencing headaches [8,14,15]. These findings are consistent with adolescents’ self-assessments of their media consumption, which show that about 18% of adolescents attribute their headaches to their media consumption [18]. On the contrary, a study investigating various types of screen-based media use did not find significant associations with headaches [19]. Similarly, an investigation on excessive media use did not establish a significant relationship with headaches [20].

Findings from previous, predominantly cross-sectional studies on the relationship between digital media use and headaches are mixed. To our knowledge, no longitudinal studies have assessed how media use is associated with features of pediatric headaches over time. This study addresses this research gap by investigating the longitudinal effect of digital media consumption on headaches that are persistent or recurrent over one year in school-aged children. Analyzing the effect of media consumption on headaches longitudinally can provide a deeper understanding of potential correlations. Since both recurrent headaches and media consumption can develop and change over time, it is important to investigate time effects, especially in the context of children’s and adolescents’ health outcomes. We hypothesize that higher media consumption will be associated with increased headache intensity and frequency, both at baseline and long term. If adverse effects are found, this would support the implementation of preventive interventions, such as in schools, which have already proven successful in other domains [21,22,23]. Early interventions could help prevent adverse health outcomes into adulthood, thus reducing the emotional and financial burden on both the affected individuals and the healthcare system.

## 2. Materials and Methods

### 2.1. Study Design and Procedure

**Figure 1:**
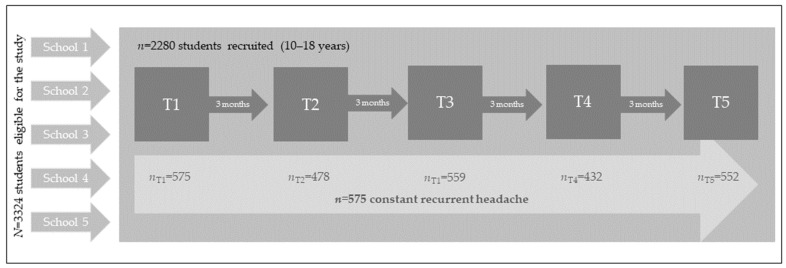
Study flow. Note. Within the CHAP project, we collected data from n = 2280 students. Only those presenting with constant recurrent headaches are considered in this work.

This study is part of the “Chronic headache in adolescence: The patient perspective on healthcare utilization” (CHAP) project. In this project, students from five secondary schools in North-Rhine Westphalia, Germany, including three school types (Gesamtschule, Realschule, Gymnasium), were recruited. Students were eligible if they were enrolled in the fifth to tenth grades and aged between 10 and 18 years. This study utilized a longitudinal design, with data collected at five measurements, each three months apart (T_1_–T_5_). Data collection took place between 2017 and 2018. The first, third, and fifth assessments were conducted on tablet computers at school. The second and fourth measurements were conducted as online surveys from home. All participants received detailed written and verbal information. And both student and parental assent/consent were required for participation. While the CHAP project also collected parental data, this study focused only on student data. For the study flow of this work, see Figure 1.

Ethics approval was granted by the committee of Witten/Herdecke University (reference number 40/2017).

### 2.2. Sample

At the five participating schools, 3324 adolescents were eligible for the study. At T_1_, *N* = 2280 students were included in the study (52.2% female; *M*_age_ = 13.0, *SD*_age_ = 1.8; see a previous CHAP publication [24] for detailed exclusion criteria). Participation at the follow-up assessments was as follows: *N*_T2_ = 1819 (53.3% female), *N*_T3_ = 2205 (52.1% female), *N*_T4_ = 1674 (53.3% female), and *N*_T5_ = 2141 students (52.4% female).

### 2.3. Measurements and Variables

At the first of the five assessments, individuals provided information on sex and age. At each measurement, students reported whether and where they had experienced pain during the last three months. In the current work, only students who reported headaches throughout the year, i.e., at almost all assessments (see the end of this paragraph for more details), were considered. Participants indicated the frequency of headaches during the past three months with the following response options: 1 = only once, 3 = about once per month, 12 = about once per week, 48 = multiple times per week, 90 = daily, 180 = always (corresponding to the number of days perceiving pain within the past three months). Chronic headaches were defined as occurring at least weekly during the past three months, consistent with former works [25,26]. To ensure the condition’s recency [24], students reporting pain were additionally asked whether they had also experienced headaches during the last four weeks.

Students reporting headaches also rated the average and strongest intensity of their headaches during the last four weeks using an 11-point numerical rating scale (NRS; 0 = no pain to 10 = strongest pain). The mean of these values was used for analysis. While items were mandatory, students could select “I am not able to answer this” instead of providing a numerical rating (coded as missing). If the average headache intensity was answered but the strongest headache intensity was missing, the mean headache intensity value was replaced with the average headache intensity value. This procedure, however, was not reversed; the strongest intensity value did not replace a missing average intensity to avoid artificially inflating the headache intensity estimate.

To assess media consumption, students reported their consumption of three different media types on both schooldays and weekends [27], following methods similar to the “Health Behaviour in School-Aged Children” (HBSC) study [28]. For this, students were asked the following: (1) “How many hours a day do you watch videos (e.g., on YouTube), DVDs, or something on TV?”; (2) hours spent playing games: “How many hours per day do you typically spend on your computer, game console, tablet, smartphone, or other electronic device playing games?”; (3) hours spent on social media/browsing the internet: “How many hours per day do you typically use your computer, tablet, or smartphone for other purposes, such as WhatsApp, Snapchat, Facebook, chatting, or surfing the web?” Response formats were as follows: not at all, about half an hour, about 1 h, about 2 h, about 3 h, about 4 h, about 5 h, about 6 h, about 7 h, or longer. Media consumption scores for schooldays and weekends were assessed separately for each type (watching videos, gaming, and social media) and aggregated to calculate average consumption per day [((consumption on schooldays × 5) + (consumption on weekends × 2))/7]. A general daily media consumption score was computed by summing the watching videos and gaming scores, following the recommendations of the HBSC study [28]. As time spent on social media overlaps with the other two media types, it was not included in the general daily media consumption score to prevent overestimation. All items on media consumption were optional.

Since our primary interest was in understanding how media consumption influences the characteristics of recurrent headaches, we focused on the subgroup of students who reported having constant recurrent headaches throughout the year. Students were categorized as having constant headaches if they reported headaches at a minimum of three out of the five assessments (T_1_–T_5_), allowing for up to two missing assessments or one assessment with no reported headaches.

### 2.4. Statistical Analysis

Descriptive statistics, including mean and standard deviations, were calculated for headache intensity, headache frequency, and general media consumption at each time point (T_1_–T_5_). The associations between media consumption, time, and headache intensity and frequency were investigated using two separate multilevel linear models (MLM) within this subgroup (R package “lme4” [29], R version 4.1.2). Model fit was significantly improved by adding the random slope. ICC > 0.1 and design effects > 2 [(1 + average cluster size − 1)*ICC] warranted the use of MLMs. The MLMs, predicting headache intensity or frequency, included fixed effects for time, sex, age, and by-subject random slopes (effect of time between individuals) and variables on media consumption. All three media consumption variables (watching television/videos, playing games, social media/browsing the internet) were centered within students (centering within clusters; cwc) and between students (centering on the grand mean; cgm). The six interactions between these media consumption variables and time were included in the model.

Due to the complexity of the model because of the inclusion of time as a random as well as fixed effect, the default “Nelder Mead” optimizer of the “lme4” package was used to avoid convergence problems due to robust optimization [29]. All analyses were conducted with R [30] utilizing RStudio [31]. For quality assurance of the planning and implementation of the study, see Supplementary Materials Table S1.

## 3. Results

### 3.1. Demographics and Descriptives

Of all *N* = 2280 participants, and across all measurements, *n* = 575 (72.9% female; *M*_age_ = 13.3, *SD*_age_ =1.86), participants reported experiencing constant headaches. The mean general media consumption over all measurements was *M* = 3.91 h per day (*SD* = 2.60), ranging from 0 to 14 h per day. The mean headache intensity, frequency, and media consumption for each time point are displayed in Table 1.

### 3.2. Multilevel Linear Models

Multilevel linear models were calculated for the sample reporting constant headaches. Separate models analyzed headache intensity and headache frequency.

#### 3.2.1. Headache Intensity

**Figure 2:**
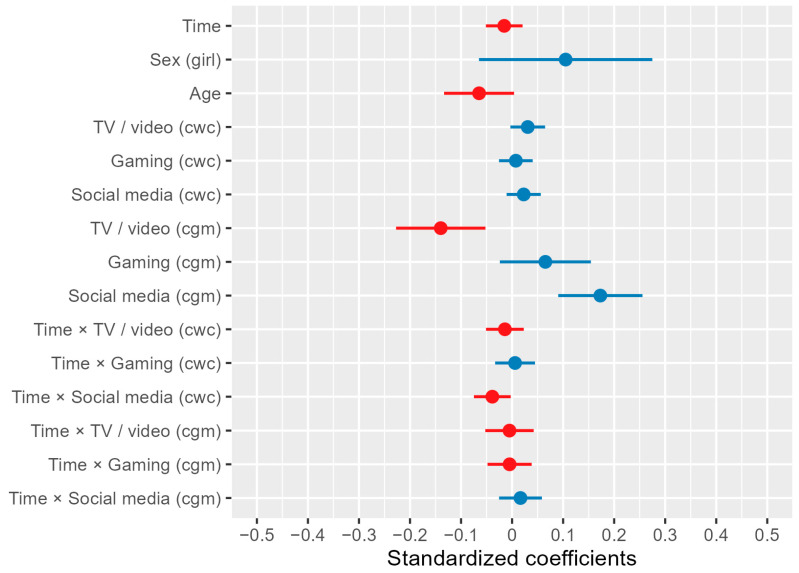
Predictors in multilevel linear model of headache intensity. Note. cwc (centering within clusters): an individual’s value at a given assessment minus their average across all assessments (within-subject centering). cgm (centering grand mean): an individual’s average across all assessments minus the overall mean of all individual averages (between-subject centering). Blue bars represent predictors that are positively associated with headache intensity (higher values of the respective variable correspond to greater headache intensity). Red bars represent predictors that are negatively associated with headache intensity (lower values of the respective variable correspond to greater headache intensity).

**Figure 3:**
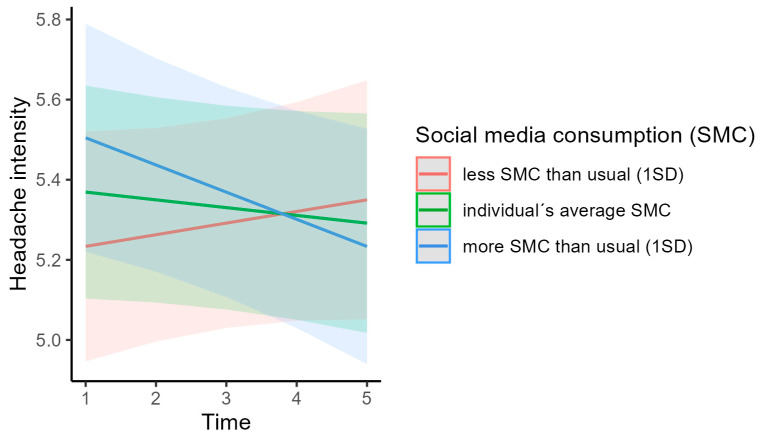
Interaction between time and social media consumption (within-subject deviation) as a predictor of headache intensity. Note. The figure illustrates the marginal effects of the interaction between time and social media consumption (within-subject) as a predictor of headache intensity. The green line represents the mean of the predictor, indicating individuals who use social media at the respective assessment with the same frequency as their average social media use across all assessments, thus those exhibiting their usual social media consumption rate at a given assessment. Red and blue lines represent one standard deviation below and above the mean, respectively. Interpretation aid: The blue line represents an individual’s increased social media consumption at a specific assessment compared with their average social media consumption over the observed year (i.e., the individual consumes social media at a higher frequency than usual at that time point). At the first assessment, this increase in social media consumption of one individual (compared with the usual consumption of that individual within the observed year) is associated with higher headache intensity compared with increases at later assessments.

The multilevel linear model assessing the association between media consumption and headache intensity revealed three statistically significant effects (Figure 2; for model details, see Appendix A). First, a student’s average television/video consumption was negatively associated with headache intensity (cgm; *t*(1989) = −3.129, *p* = 0.002). This means that students with recurrent headaches who watched more television/videos tended to report less intense headaches. Second, a student’s average social media consumption was positively correlated with headache intensity (cgm; *t*(1989) = 4.109, *p* < 0.001). This indicates that students consuming social media more frequently reported higher headache intensity. Third, the interaction between time and social media consumption at a specific assessment was negatively associated with headache intensity (cwc; *t*(1989) = −2.105, *p* = 0.035). This suggests that students who consumed more social media than usual early in the school year were more likely to report higher pain intensity than students who consumed more social media later in the school year. Conversely, students who consumed social media less than usual at the beginning of the school year were more likely to report lower headache intensity than students who consumed less social media than usual at the end of the school year (Figure 3).

#### 3.2.2. Headache Frequency

**Figure 4:**
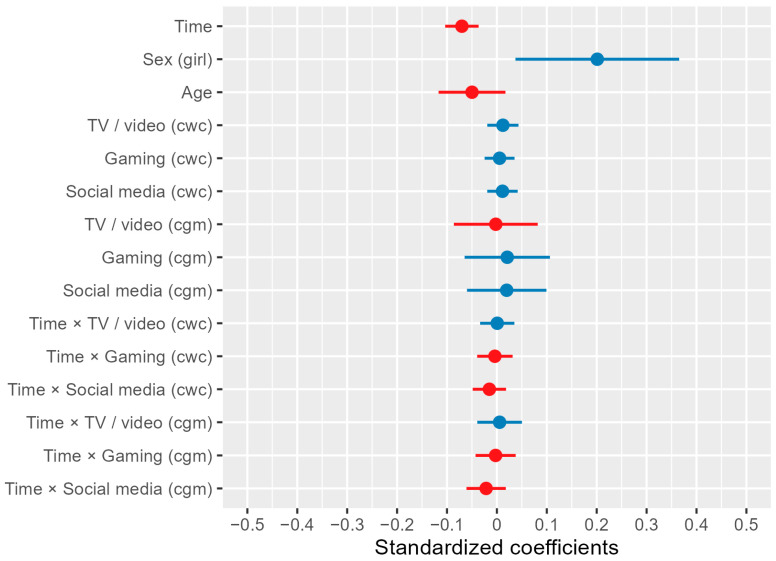
Predictors in multilevel linear model of headache frequency. Note. cwc (centering within clusters): the individual’s value per assessment minus their mean across all assessments (within-subject centering). cgm (centering grand mean): the individual’s average across all assessments minus the mean of all individuals’ means across all assessments (between-subject centering). The blue bars represent predictors that are positively associated with headache frequency. Conversely, the red bars represent predictors that are negatively associated with headache frequency.

In the multilevel model investigating the association between media consumption and headache frequency, the main effects of time and sex were statistically significant. Time was negatively associated with headache frequency (*t*(2351) = −4.105, *p* < 0.001), indicating that headache frequency decreased over time. Furthermore, sex was associated with headache frequency (*t*(2351) = 2.405, *p* = 0.016), with girls reporting more frequent headaches than boys. Media consumption was not significantly associated with headache frequency (Figure 4; for model details, see Appendix B).

## 4. Discussion

In the present study, the association between media consumption and headache intensity and frequency over time was assessed in a sample of adolescents reporting recurrent headaches throughout the year. This subsample accounted for approximately 25% of the total sample from the general student population in the German CHAP study. This is in line with previous works that also found high headache prevalence in school samples [16,17]. More girls than boys reported experiencing constant headaches throughout the observed year, which aligns with previous studies reporting that girls are at higher risk for chronic pain conditions [24,32,33]. The average screen time in our sample was four hours per day, comparable to other pre-pandemic assessments [34,35]. Data were collected shortly before the COVID-19 pandemic, during which there was an increase in media consumption among youths [18].

Regarding the association between media consumption and headache intensity, we found statistically significant results for television/video consumption and social media usage. Of interest, the deviation from a person’s average television/video consumption and the mean of all individuals in this group was negatively associated with headache intensity. This suggests that adolescents reporting recurrent headaches who watch television/videos more frequently than the average adolescent with recurrent headaches tend to report lower headache intensity. This finding could result in the misleading interpretation that watching television could be a protective factor against higher headache intensity. However, one needs to consider that the major chronic headache types among youths are tension-type headaches and migraines [36,37,38]. One diagnostic criterion for migraine is moderate to high headache intensity, whereas tension-type headaches typically present with mild to moderate intensity [39]. Consequently, individuals reporting high headache intensity in our study are more likely to be suffering from migraines rather than tension-type headaches. A typical concomitant feature of migraine is the avoidance of strong light stimuli (“photophobia”), such as the light from televisions [39]. Thus, it is reasonable to assume that adolescents reporting higher screen time for watching television or videos were more likely affected by tension-type headaches, which are not typically accompanied by photophobia. If this interpretation holds true, media consumption could be influenced by the type and features of chronic headache conditions rather than chronic headache conditions being directly influenced by media consumption. This interpretation is in line with a Turkish study, which found that recurrent headaches were associated with lower levels of internet addiction, suggesting that media devices are avoided because they are associated with migraine [40]. It should be noted, however, that we did not consider the type of recurrent headache in our analysis, as this would have introduced circular reasoning—headache intensity itself is a diagnostic criterion for migraine.

In our study, we looked at a sample of adolescents reporting constant recurrent headaches without remission, suggesting that certain factors are sustaining the condition. Adolescents suffering from tension-type headaches are advised to adapt their lifestyle, such as by engaging in regular physical activities or limiting the use of electronic devices [41,42]. Given that only a small proportion of adolescents affected by chronic pain seek medical consultation [43], it is likely that many are unaware of these recommendations and, as a result, spend more time consuming media. This indicates the need for greater efforts within our society to shed light on chronic pain conditions and how to manage them effectively.

Regarding social media consumption, the deviation of a person’s mean social media usage from the average usage of all adolescents was positively correlated with headache intensity. This suggests that individuals who consume social media more frequently than the average youth report higher headache intensity. As with television/video consumption, this finding does not allow causal interpretation. However, we made a further, interesting observation that may help explain this relationship. The interaction between time and the deviation in an individual’s social media consumption at a certain point in time, relative to their average social media consumption over the year (within-subject deviation), was negatively associated with headache intensity. This indicates that social media consumption at the beginning of the assessment period that was at a higher level than throughout the rest of the year was associated with increased headache intensity, compared with consuming social media at the end of the assessment at a higher level than throughout the previous assessments of the observed year. This finding is somewhat difficult to interpret. One possible explanation is that social media usage affects headache intensity in the long term. If individuals reported high social media consumption at the start of the observed year, it is likely that their social media use had been elevated prior to the assessment as well. Assuming that prolonged social media usage affects headache intensity, this might explain why these individuals had higher headache intensity compared to those who only increased their social media usage at the end of the year. This explanation is supported by the finding that individuals who reported higher social media usage throughout the year, relative to their peers, also reported higher headache intensity. However, it is important to note that this interpretation is speculative and needs further verification in future research.

If social media consumption does indeed affect headache intensity in the long run, it is likely that there are underlying moderators or mediators involved. For example, an increase in social media consumption might be associated with withdrawal from daily activities for various reasons. Furthermore, burdensome content and online bullying on social media platforms can contribute to depressive moods [9]. According to the fear-avoidance model of chronic pain, withdrawal can exacerbate chronic pain conditions [44]. Thus, information about increased social media usage could provide helpful context when treating chronic pain. First, clinicians and psychotherapists who detect an intra-individual increase in social media consumption can use this as a starting point for recommending lifestyle changes and providing psychoeducation. For example, patients with chronic pain might withdraw from social activities to avoid potential pain triggers. To compensate, they may turn to social media more often and adopt a more passive lifestyle. This shift in behavior could make them focus more on their pain. Thus, they experience pain more often, their fear of pain increases, and the vicious cycle continues [44]. Second, raising awareness of the potential risks associated with increased social media use could empower individuals to manage their condition more effectively. For instance, app-based interventions could be implemented, similar to those used in the treatment of bipolar disorder, where changes in smartphone usage patterns (e.g., frequency of social communication) serve as indicators of impending manic or depressive episodes [45]. The involvement of mediators and moderators may also help explain the mixed results on the association between media consumption and headache conditions. Future research should explore these factors further to clarify the mechanisms at play.

Why is it that among the three analyzed media consumption types, gaming was not associated with headache intensity? A key feature of gaming, as opposed to other media types, is its active nature. When gaming, individuals must actively analyze and respond to situations, which provides significant distraction from pain—an effect often advised to seek when treating chronic pain conditions [46]. This distracting effect has been demonstrated in virtual reality, which likely shares similarities with gaming experiences [47]. In contrast, the consumption of social media or watching television/videos is more passive and, therefore, may not offer the same level of distraction. Even if this assumption holds true, recommendations regarding gaming cannot be drawn from our data, as we did not observe significant associations with decreased headache intensity or frequency. Similar to social media, increased gaming time may indicate withdrawal from other daily activities. However, gaming might offer a buffering effect (e.g., stronger distraction or less burdensome content) that prevents the pain condition from worsening.

Regarding the association between media consumption and headache frequency, we found no significant associations with any media type. This may support the hypothesis that pain is not directly linked with the duration of media consumption but rather that media consumption may serve as a mediator or moderator for factors that influence the pain experience.

However, we did observe that headache frequency decreased over time and that girls reported a higher frequency of headaches compared to boys. This decrease in headache frequency over time might reflect remission, as has been previously linked to psychological factors such as the use of coping strategies [48].

As opposed to our initial hypothesis, there were only minor associations between extended media consumption time and headache features. This is in line with a recent publication that also did not find significant correlations between headache frequency and screen exposure in adolescents [49], as well as a longitudinal study that found no associations between pain and screen time [50]. Similarly, longitudinal studies investigating the relationship between screen time and mental health problems or attention-deficit/hyperactivity disorder have also found no or only small effects [51,52]. Most studies reporting positive associations between headache and media exposure are based on cross-sectional designs, which only assess data from a single assessment and do not capture the development of symptoms over time [8,14]. Thus, it was crucial to present results from multiple assessments over time regarding the association between media consumption and chronic headache conditions. Considering the results of our work, we propose that time spent consuming media is not directly associated with headache features but may instead serve as an indicator of other aspects that promote or sustain pain conditions (e.g., withdrawal from activities). To further investigate risk factors, future research should focus on analyzing the content of social media rather than the amount of time spent using it [9]. Additionally, it would be valuable to examine the impact of how the media portrays pain, as other works indicate that children’s media often lacks sufficient role models for effective pain management [53,54].

It must be taken into account that if data collection had taken place post-pandemic, very different results could have emerged, as other studies have revealed positive associations between screen time and social media use during the COVID-19 pandemic [18]. Studies conducted during or after the pandemic found that screen time usage doubled during this period, from 4 to 8 h per day [34,55]. Particularly regarding social media, where significant results emerged in the current study, a lot has changed during the last few years. Social media platforms like Instagram or TikTok have gained immense popularity, and children and adolescents have substantially increased their use of these platforms. Therefore, longitudinal data collected before, during, and after the pandemic would be invaluable to compare the current findings on media use and its association with headache frequency and intensity, as well as to investigate the long-term changes that occurred during the COVID-19 pandemic.

### Strengths and Limitations

The current study focuses on how time spent consuming media was associated with the features of ongoing headache conditions. A limitation of this study is that we could not account for the onset or remission of headaches. Including this information would have enabled the comparison between pain-free and pain episodes, providing additional insights into the long-term effects of media usage on headache characteristics. Furthermore, we did not account for fluctuations in headache intensity, despite chronic pain conditions bringing numerous biopsychosocial factors [56]. It should be kept in mind that media consumption is only one of many environmental factors associated with chronic pain. As our results indicate, moderating mechanisms could be at play, and the inclusion of other biopsychosocial variables associated with headache conditions in childhood and adolescence could have enhanced our understanding of potential interdependencies. Variables such as the onset and remission of headaches should be prioritized in future research.

Another limitation of our study is that we only analyzed the time spent consuming media, not its content. Different types of media content may elicit different effects on the consumer, such as fostering social pressure versus empowerment or evoking positive versus negative emotions [9]. Including this information could have made the effects of media consumption on pain more evident.

This work is among the first to describe longitudinal data on the association between media use and headache features. It is enhanced by a large sample size and the inclusion of different kinds of media consumption. Furthermore, the data were collected from the general underage population rather than from a clinical population. This enables a more representative sample of this age group. This is particularly important, as few individuals affected by chronic pain seek medical consultation. These individuals would have been missed in a clinical sample [43].

## 5. Conclusions

In the current study, we found that headache frequency decreased over time and that girls reported higher headache frequency than boys. We also observed that individuals with recurrent headaches reported lower headache intensity when watching more television. We interpreted that these individuals might not suffer from migraine, which typically involves the avoidance of light stimuli such as television. Furthermore, higher social media consumption was associated with worse headache intensity—presumably in the long term. No associations were found between headache frequency and media consumption. Overall, our results suggest that there is no direct negative association of extended time spent consuming media with headache intensity or frequency. However, monitoring the duration of social media consumption may provide useful diagnostic information for managing headache conditions.

## Figures and Tables

**Table 1:** Descriptive statistics for headache features and media consumption of students with constant headaches.

	T1 *M (SD)*	T2 *M (SD)*	T3 *M (SD)*	T4 *M (SD)*	T5 *M (SD)*
Constant Headache Sample: *N* = 575					
Headache Intensity ^1^	5.67 (1.74)	5.51 (1.70)	5.40 (1.86)	5.58 (1.92)	5.53 (1.81)
Headache Frequency ^2^	31.0 (30.9)	24.1 (23.8)	24.2 (27.6)	24.9 (28.2)	23.8 (28.6)
Media Consumption ^3^	4.03 (2.80)	3.79 (2.33)	4.01 (2.64)	3.75 (2.42)	3.92 (2.71)

^1^ Headache intensity was measured on a 0–10 Numeric Rating Scale (10 = maximum intensity). ^2^ Headache Frequency is displayed in days during the last three months (0–90 days or 180 = always). ^3^ Media consumption (TV/video + gaming) was assessed in hours per day.

## Data Availability

The data presented in this study are available upon request from the corresponding author (J.W.). The data are not publicly available because, as described in the consent forms and thus, due to legal reasons, they may only be analyzed with legitimate interest.

## Supplementary Materials

The following supporting information can be downloaded at: https://www.mdpi.com/article/10.3390/children11121549/s1, Table S1: STROBE Statement—checklist of items that should be included in reports of observational studies.

## Appendix A. Multilevel Model Headache Intensity with Media Variables

**Parameter**	**Coefficient**	**SE**	**CI**	**CI_low_**	**CI_high_**	***t***	**df_error_**	***p***	**Effects**	**Group**
Intercept	−0.11	0.07	0.95	−0.25	0.03	−1.581	1989	0.114	fixed	
Time	−0.02	0.02	0.95	−0.05	0.02	−0.839	1989	0.402	fixed	
Sex (girl)	0.10	0.09	0.95	−0.06	0.27	1.212	1989	0.226	fixed	
Age	−0.06	0.03	0.95	−0.13	0.00	−1.849	1989	0.065	fixed	
TV/video (cwc)	0.03	0.02	0.95	−0.00	0.06	1.765	1989	0.078	fixed	
Gaming (cwc)	0.01	0.02	0.95	−0.03	0.04	0.438	1989	0.661	fixed	
Social media (cwc)	0.02	0.02	0.95	−0.01	0.06	1.332	1989	0.183	fixed	
TV/video (cgm)	−0.14	0.04	0.95	−0.23	−0.05	−3.129	1989	0.002	fixed	
Gaming (cgm)	0.07	0.05	0.95	−0.02	0.15	1.434	1989	0.152	fixed	
Social media (cgm)	0.17	0.04	0.95	0.09	0.26	4.109	1989	<0.001	fixed	
Time × TV/video (cwc)	−0.01	0.02	0.95	−0.05	0.02	−0.747	1989	0.455	fixed	
Time × Gaming (cwc)	0.01	0.02	0.95	−0.03	0.04	0.293	1989	0.769	fixed	
Time × social media (cwc)	−0.04	0.02	0.95	−0.07	−0.00	−2.105	1989	0.035	fixed	
Time × TV/video (cgm)	−0.01	0.02	0.95	−0.05	0.04	−0.210	1989	0.834	fixed	
Time × Gaming (cgm)	−0.00	0.02	0.95	−0.05	0.04	−0.221	1989	0.825	fixed	
Time × Social Media (cgm)	0.02	0.02	0.95	−0.03	0.06	0.770	1989	0.441	fixed	
SD (Intercept)	0.69		0.95						random	individual
SD (Time)	0.20		0.95						random	individual
Cor (Intercept~Time)	0.14		0.95						random	individual
SD (Observations)	0.68		0.95						random	Residual
Note. cwc (centering within clusters): the individual’s value per assessment minus the individual’s mean across all assessments (within-subject centering). cgm (centering grand mean): the individual’s mean across all assessments minus the overall mean of all individuals’ means across all assessments (between-subject centering).										

## Appendix B. Multilevel Model Headache Frequency with Media Variables

**Parameter**	**Coefficient**	**SE**	**CI**	**CI_low_**	**CI_high_**	***t***	**df_error_**	***p***	**Effects**	**Group**
Intercept	−0.15	0.07	0.95	−0.29	−0.02	−2.190	2351	0.029	fixed	
Time	−0.07	0.02	0.95	−0.10	−0.04	−4.105	2351	<0.001	fixed	
Sex (girl)	0.20	0.08	0.95	0.04	0.37	2.405	2351	0.016	fixed	
Age	−0.05	0.03	0.95	−0.12	0.02	−1.459	2351	0.145	fixed	
TV/video (cwc)	0.01	0.02	0.95	−0.02	0.04	0.755	2351	0.451	fixed	
Gaming (cwc)	0.01	0.02	0.95	−0.02	0.04	0.350	2351	0.726	fixed	
Social media (cwc)	0.01	0.02	0.95	−0.02	0.04	0.720	2351	0.472	fixed	
TV/video (cgm)	−0.00	0.04	0.95	−0.09	0.08	−0.051	2351	0.959	fixed	
Gaming (cgm)	0.02	0.04	0.95	−0.06	0.11	0.477	2351	0.634	fixed	
Social media (cgm)	0.02	0.04	0.95	−0.06	0.10	0.485	2351	0.628	fixed	
Time × TV/video (cwc)	0.00	0.02	0.95	−0.03	0.04	0.043	2351	0.966	fixed	
Time × Gaming (cwc)	−0.00	0.02	0.95	−0.04	0.03	−0.222	2351	0.824	fixed	
Time × social media (cwc)	−0.02	0.02	0.95	−0.05	0.02	−0.884	2351	0.377	fixed	
Time × TV/video (cgm)	0.01	0.02	0.95	−0.04	0.05	0.241	2351	0.810	fixed	
Time × Gaming (cgm)	−0.00	0.02	0.95	−0.04	0.04	−0.125	2351	0.900	fixed	
Time × Social Media (cgm)	−0.02	0.02	0.95	−0.06	0.02	−1.071	2351	0.284	fixed	
SD (Intercept)	0.69		0.95						random	individual
SD (Time)	0.22		0.95						random	individual
Cor (Intercept~Time)	0.02		0.95						random	individual
SD (Observations)	0.68		0.95						random	Residual
Note. cwc (centering within clusters): the individual’s value per assessment minus the individual’s mean across all assessments (within-subject centering). cgm (centering grand mean): the individual’s mean across all assessments minus the overall mean of all individuals’ means across all assessments (between-subject centering).										
