# Supplementary material for: Is There a Long-Term Link Between Digital Media Use and Adolescent Headaches? A Longitudinal School-Based Study
Source: Children (Basel). 2024 Dec 20;11(12):1549. doi: 10.3390/children11121549 (PMC11727571; doi:10.3390/children11121549)
Supplement: Supplementary file 1 [file children-11-01549-s001.zip › children-3363433-supplementary.pdf]

Table S1. STROBE Statement—checklist of items that should be included in reports of observational studies

	Item No.	Recommendation	Page No.	Relevant text from manuscript
**Title and abstract**	1	(*a*) Indicate the study's design with a commonly used term in the title or the abstract	1	Title: A longitudinal school-based study.
		(*b*) Provide in the abstract an informative and balanced summary of what was done and what was found	1	In total, *N*=575 (72.9% female; M_{age}=13.3, SD_{age} = 1.86) children and adolescents reporting consistent headaches across all assessments were analyzed. Multilevel linear modelling was used to assess the relationships between media use and headache frequency and intensity over time. There were only minor associations between media use and headache intensity or frequency. Notably, only high social media usage was linked with worse headache intensity ($t(1989)=4.109$, $p<.001$).
**Introduction**				
Background/rationale	2	Explain the scientific background and rationale for the investigation being reported	1-2	Findings from previous, predominantly cross-sectional studies on the relationship between digital media use and headaches are mixed. To our knowledge, no longitudinal studies have assessed how

				media use is associated with features of pediatric headaches over time.
Objectives	3	State specific objectives, including any prespecified hypotheses	2	This study addresses this research gap by investigating the longitudinal effect of digital media consumption on headaches that are persistent or recurrent over one year in school-aged children. We hypothesize that higher media consumption will be associated with increased headache intensity and frequency, both at baseline and long-term.
**Methods**				
Study design	4	Present key elements of study design early in the paper	2	The study utilized a longitudinal design, with data collected at five measurements, each three months apart (T_1-T_5).
Setting	5	Describe the setting, locations, and relevant dates, including periods of recruitment, exposure, follow-up, and data collection	2	In this project, students from five secondary schools in North-Rhine Westphalia, Germany, including three school types (Gesamtschule, Realschule, Gymnasium), were recruited. Students were eligible if they were enrolled in the fifth to tenth grades and aged between 10 and 18 years. The study utilized a longitudinal design,

				with data collected at five measurements, each three months apart (T_1-T_5). Data collection took place between 2017 and 2018. The first, third, and fifth assessments were conducted on tablet computers at school. The second and fourth measurements were conducted as online surveys from home.
Participants	6	(*a*) *Cohort study*—Give the eligibility criteria, and the sources and methods of selection of participants. Describe methods of follow-up<br>*Case-control study*—Give the eligibility criteria, and the sources and methods of case ascertainment and control selection. Give the rationale for the choice of cases and controls<br>*Cross-sectional study*—Give the eligibility criteria, and the sources and methods of selection of participants	2 & 4	Students were eligible if they were enrolled in the fifth to tenth grades and aged between 10 and 18 years.<br>Since our primary interest was in understanding how media consumption influences the characteristics of recurrent headaches, we focused on the subgroup of students who reported having constant recurrent headaches throughout the year. Students were categorized as having constant headaches if they reported headaches at a minimum of three out of the five assessments (T_1-T_5), allowing for up to two missing assessments or one assessment with no reported headaches.

		(*b*) *Cohort study*—For matched studies, give matching criteria and number of exposed and unexposed<br>*Case-control study*—For matched studies, give matching criteria and the number of controls per case		
Variables	7	Clearly define all outcomes, exposures, predictors, potential confounders, and effect modifiers. Give diagnostic criteria, if applicable	3-4	In the current work, only students who reported headaches throughout the year, i.e. at almost all assessments […] were considered. Participants indicated the *frequency* of headaches during the past three months […]. Students reporting headaches also rated the average and strongest *intensity* of their headaches during the last four weeks, using an 11-point numerical rating scale (NRS; 0=*no pain* to 10=*strongest pain*). To assess media consumption, students reported their consumption of three different media types on both schooldays and weekends, following methods similar to the "Health Behaviour in School-Aged Children" (HBSC) study.
Data sources/ measurement	8*	For each variable of interest, give sources of data and details of methods of assessment (measurement). Describe comparability of assessment methods if there is more than one group	3-4	Participants indicated the *frequency* of headaches during the past three months with the following response options: 1=*only once*, 3=*about once per*

month, 12=*about once per week*, 48=*multiple times per week*, 90=*daily*, 180=*always* (corresponding to the number of days perceiving pain within the past three months).
Students reporting headaches also rated the average and strongest *intensity* of their headaches during the last four weeks, using an 11-point numerical rating scale (NRS; 0=*no pain* to 10=*strongest pain*). The mean of these values was used for analysis. While items were mandatory, students could select "*I am not able to answer this*" instead of providing a numerical rating (coded as missing).
To assess media consumption, students reported their consumption of three different media types on both schooldays and weekends, following methods similar to the "Health Behaviour in School-Aged Children" (HBSC) study. For this, students were asked: (1) "How many hours a day do you watch videos (e.g. on YouTube), DVDs or something

on TV?”; (2) hours spent playing games: “How many hours per day do you typically spend on your computer, game console, tablet, smartphone, or other electronic device playing games?”; (3) hours spent on social media/browsing the internet: “How many hours per day do you typically use your computer, tablet, or smartphone for other purposes, such as WhatsApp, Snapchat, Facebook, chatting, or surfing the web?”. Response formats were as follows: *not at all*, *about half an hour*, *about an hour*, *about two hours*, *about 3 hours*, *about 4 hours*, *about 5 hours*, *about 6 hours*, *about seven hours or longer*. Media consumption scores for schooldays and weekends were assessed separately for each type (watching videos, gaming, and social media) and aggregated to calculate average consumption per day [((consumption on schooldays * 5) + (consumption on weekends * 2)) / 7]. A general daily media consumption score was

				computed by summing the watching videos and gaming scores, following the recommendations of the HBSC study. As time spent on social media overlaps with the other two media types, it was not included in the general daily media consumption score to prevent overestimation. All items on media consumption were optional.
Bias	9	Describe any efforts to address potential sources of bias	-	-
Study size	10	Explain how the study size was arrived at	3-4	(see also Figure 1); At the five participating schools, 3324 adolescents were eligible for the study. At T_1, *N*=2280 students were included in the study. Of all *N*=2280 participants and across all measurements, *n*=575 (72.9% female; M_{age}=13.3, SD_{age} =1.86) participants reported experiencing constant headaches.

Continued on next page

Quantitative variables	11	Explain how quantitative variables were handled in the analyses. If applicable, describe which groupings were chosen and why	3-4	Participants indicated the *frequency* of headaches during the past three months with the following response options: 1=*only once*, 3=*about once per month*, 12=*about once per week*, 48=*multiple times per week*, 90=*daily*, 180=*always* (corresponding to the number of days perceiving pain within the past three months).<br>Students reporting headaches also rated the average and strongest *intensity* of their headaches during the last four weeks, using an 11-point numerical rating scale (NRS; 0=*no pain* to 10=*strongest pain*). The mean of these values was used for analysis.<br>To assess media consumption, students reported their consumption of three different media types on both schooldays and weekends […] Media consumption scores for schooldays and weekends were assessed separately for each type (watching videos, gaming, and social media) and aggregated to calculate average consumption per day [((consumption on schooldays * 5) + (consumption on weekends * 2)) / 7]. A general daily media consumption score was computed

				by summing the watching videos and gaming scores, following the recommendations of the HBSC study. As time spent on social media overlaps with the other two media types, it was not included in the general daily media consumption score to prevent overestimation.
Statistical methods	12	(*a*) Describe all statistical methods, including those used to control for confounding	4	Descriptive statistics, including mean and standard deviations, were calculated for headache intensity, headache frequency, and general media consumption at each time point (T_1-T_5). The associations between media consumption, time, and headache intensity and frequency were investigated using two separate multilevel linear models (MLM) within this subgroup. The MLMs, predicting headache intensity or frequency, included fixed effects for time, sex, age, and by-subject random slopes (effect of time between individuals) and variables on media consumption. All three media consumption variables (watching television/videos, playing games, social media/browsing the internet) were centered within students (centering within clusters; cwc) and

			between students (centering on the grand mean; cgm). The six interactions between these media consumption variables and time were included in the model. Due to the complexity of the model by including time as a random as well as fixed effect, the default "Nelder Mead" optimizer of the *lme4* package has been used to avoid convergence problems due to robust optimization.
	(*b*) Describe any methods used to examine subgroups and interactions	4	The MLMs, predicting headache intensity or frequency, included fixed effects for time, sex, age, and by-subject random slopes (effect of time between individuals) and variables on media consumption. All three media consumption variables (watching television/videos, playing games, social media/browsing the internet) were centered within students (centering within clusters; cwc) and between students (centering on the grand mean; cgm). The six interactions between these media consumption variables and time were included in the model.
	(*c*) Explain how missing data were addressed	3	If average headache intensity was answered but the strongest headache intensity was missing, the

				mean headache intensity value was replaced with the average headache intensity value.
		(*d*) *Cohort study*—If applicable, explain how loss to follow-up was addressed<br>*Case-control study*—If applicable, explain how matching of cases and controls was addressed<br>*Cross-sectional study*—If applicable, describe analytical methods taking account of sampling strategy	-	-
		(*e*) Describe any sensitivity analyses	-	-
**Results**				
Participants	13*	(a) Report numbers of individuals at each stage of study—eg numbers potentially eligible, examined for eligibility, confirmed eligible, included in the study, completing follow-up, and analysed	3 & 4	At the five participating schools, 3324 adolescents were eligible for the study. At T_1, *N*=2280 students were included in the study (52.2% female; M_{age} = 13.0, SD_{age} = 1.8; see a previous CHAP publication for detailed exclusion criteria). Participation at the follow-up assessments was as follows: N_{T2}=1819 (53.3% female), N_{T3}=2205 (52.1% female), N_{T4}=1674 (53.3% female), and N_{T5}=2141 students (52.4% female). Of all *N*=2280 participants and across all measurements, *n*=575 (72.9% female; M_{age}=13.3, SD_{age} =1.86) participants reported experiencing constant headaches.
		(b) Give reasons for non-participation at each stage	-	-
		(c) Consider use of a flow diagram	3	Figure 1
Descriptive data	14*	(a) Give characteristics of study participants (eg demographic, clinical, social) and information on exposures and potential confounders	4-5	Of all *N*=2280 participants and across all measurements, *n*=575

				(72.9% female; M_{age}=13.3, SD_{age} =1.86) participants reported experiencing constant headaches. The mean general media consumption over all measurements was *M*=3.91 hours per day (*SD*=2.60), ranging from 0 to 14 hours per day.
		(b) Indicate number of participants with missing data for each variable of interest	-	-
		(c) *Cohort study*—Summarise follow-up time (eg, average and total amount)	-	-
Outcome data	15*	*Cohort study*—Report numbers of outcome events or summary measures over time	4-5	Table 1
		Case-control study—Report numbers in each exposure category, or summary measures of exposure		
		Cross-sectional study—Report numbers of outcome events or summary measures		
Main results	16	(*a*) Give unadjusted estimates and, if applicable, confounder-adjusted estimates and their precision (eg, 95% confidence interval). Make clear which confounders were adjusted for and why they were included	12-13	Appendices A & B
		(*b*) Report category boundaries when continuous variables were categorized	-	-
		(*c*) If relevant, consider translating estimates of relative risk into absolute risk for a meaningful time period	-	-

Continued on next page

Other analyses	17	Report other analyses done—eg analyses of subgroups and interactions, and sensitivity analyses	-	-
**Discussion**				
Key results	18	Summarise key results with reference to study objectives	8-10	Regarding the association between media consumption and headache intensity, we found statistically significant results for television/video consumption and social media usage. Of interest, the deviation from a person´s average television/video consumption and the mean of all individuals in this group was negatively associated with headache intensity. However, one needs to consider that the major chronic headache types among youths are tension- type headache and migraine.<br>Regarding social media consumption, the deviation of a person´s mean social media usage from the average usage of all adolescents was positively correlated with headache intensity. This suggests that individuals who consume social media more frequently than the average youth report higher headache intensity. The interaction between time and the deviation in an individual's social media consumption at a certain point in time, relative to

their average social media consumption over the year (within-subject deviation), was negatively associated with headache intensity. One possible explanation is that social media usage affects headache intensity long-term.

Why is it that among the three analyzed media consumption types, gaming was not associated with headache intensity? A key feature of gaming, as opposed to other media types, is its active nature. When gaming, individuals must actively analyze and respond to situations, which provides significant distraction from pain – an effect often advised to seek when treating chronic pain conditions.

Regarding the association between media consumption and headache frequency, we found no significant associations with any media type. However, we did observe that headache frequency decreased over time, and that girls reported a higher frequency of headaches compared to boys.

As opposed to our initial hypothesis, there were only minor associations between extended

				media consumption time and headache features.
Limitations	19	Discuss limitations of the study, taking into account sources of potential bias or imprecision. Discuss both direction and magnitude of any potential bias	11	A limitation of this study is that we could not account for the onset or remission of headaches. Including this information would have enabled the comparison between pain-free and pain episodes, providing additional insights into the long-term effects of media usage on headache characteristics. Furthermore, we did not account for fluctuations in headache intensity, despite chronic pain conditions bring numerous biopsychosocial factors. Another limitation of our study is that we only analyzed the time spent consuming media, not its content.
Interpretation	20	Give a cautious overall interpretation of results considering objectives, limitations, multiplicity of analyses, results from similar studies, and other relevant evidence	10-11	As opposed to our initial hypothesis, there were only minor associations between extended media consumption time and headache features. This is in line with a recent publication that also did not find significant correlations between headache frequency and screen exposure in adolescents, as well as a longitudinal study that found no associations between pain and screen time. Similarly, longitudinal studies investigating

				the relationship between screen time and mental health problems or attention-deficit/hyperactivity disorder have also found no or only small effects [51,52]. Most studies reporting positive associations between headache and media exposure are based on cross-sectional designs, which only assess data from a single assessment and do not capture the development of symptoms over time. Considering the results of our work, we propose that time spent consuming media is not directly associated with headache features but may instead serve as an indicator of other aspects that promote or sustain pain conditions (e.g. withdrawal from activities).
Generalisability	21	Discuss the generalisability (external validity) of the study results	10-11	Most studies reporting positive associations between headache and media exposure are based on cross-sectional designs, which only assess data from a single assessment and do not capture the development of symptoms over time. Thus, it was crucial to present results from multiple assessments over time regarding the association between media consumption and chronic headache conditions. Considering

				the results of our work, we propose that time spent consuming media is not directly associated with headache features but may instead serve as an indicator of other aspects that promote or sustain pain conditions (e.g. withdrawal from activities). It must be taken into account that if data collection had taken place post-pandemic, very different results could have emerged, as other studies have revealed positive associations between screen time and social media use during the COVID-19 pandemic.
**Other information**				
Funding	22	Give the source of funding and the role of the funders for the present study and, if applicable, for the original study on which the present article is based	12	This research was funded by the German Federal Ministry of Education and Research, grant number 01GY1615.

*Give information separately for cases and controls in case-control studies and, if applicable, for exposed and unexposed groups in cohort and cross-sectional studies.

Note: An Explanation and Elaboration article discusses each checklist item and gives methodological background and published examples of transparent reporting. The STROBE checklist is best used in conjunction with this article (freely available on the Web sites of PLoS Medicine at http://www.plosmedicine.org/, Annals of Internal Medicine at http://www.annals.org/, and Epidemiology at http://www.epidem.com/). Information on the STROBE Initiative is available at www.strobe-statement.org.
